# Comparison of Superchilling and Supercooling on Extending the Fresh Quality of Beef Loin

**DOI:** 10.3390/foods11182729

**Published:** 2022-09-06

**Authors:** Honggyun Kim, Geun-Pyo Hong

**Affiliations:** Department of Food Science and Biotechnology, Sejong University, Seoul 05006, Korea

**Keywords:** subzero temperature, superchilling, supercooling, preservation, beef loin, freshness

## Abstract

This study compared the effects of superchilling and supercooling preservations for 15 days on the freshness and quality characteristics of beef loin. Beef freshness was evaluated by total aerobic count (TAC), total volatile basic nitrogen (TVB-N), and thiobarbituric acid reactive substances (TBARS), and instrumental color, drip loss, cooking loss, and texture profile analysis (TPA) were determined as quality parameters. All assays were compared with fresh control and normal chilling conditions (4 °C). The mean preservation temperatures of superchilling and supercooling were −3.9 °C and −2.1 °C, respectively. The freshness parameters indicated that both superchilling and supercooling extended the freshness of beef loin for 15 days, while chilled beef could not maintain the standard of freshness conditions. For quality parameters, there was no difference between the control and supercooling treatments, whereas superchilling exhibited higher drip loss and toughness compared to the control (*p* < 0.05). Therefore, this study demonstrated that supercooling was the best preservation technique to extend the freshness and quality of beef loin, but superchilling was not suitable to guarantee the quality of beef.

## 1. Introduction

Recently, consumers’ demands for preservation technology have shifted to maintain the original freshness of foods for weeks rather than extending the edible condition for very long periods [1]. Meat is a highly perishable food; hence, low-temperature preservation has been essential for meat storage. Due to postmortem biochemical and microbial metabolism, meat undergoes rapid physicochemical changes during normal chilled storage (~4 °C), which allows only limited storage periods [2]. Therefore, meat freezing has been commonly applied to extend shelf life for more than 6 months. Nevertheless, quality deteriorations involved in the physical tissue damage of meat were inevitably manifested even if ultrafast freezing was applied [3], and current novel techniques have been focused on preservation at subzero temperatures without the freezing of meat [2,4]. Supercooling is considered as a representative novel technique that can extend the shelf life and wholesomeness of meat by 2–3 weeks [1,5].

Supercooling preservation is defined as a process to store foods at temperatures below their freezing point without the occurrence of freezing [2]. Research to develop supercooling preservation has been carried out for a long time; nevertheless, the generation of stable supercooling conditions has not been successfully obtained due to its quasi-stable nature [6]. High pressure and electromagnetic fields have been intensively investigated as potential techniques of supercooling preservation; nevertheless, utilization of these technologies is limited in lab-scale tests [7,8]. Recently, a stepwise cooling algorithm which applied cycles of the regular temperature declines was introduced as a feasible supercooling storage of meat and fish. This method is based on the probability of increasing the extent of supercooling by controlling the cooling rate [6,9]. Studies indicated that supercooling storage could extend the freshness of meat and fish up to 2–3 weeks compared to the 3~5 days of commercially chilled counterparts [1,10]. However, this method showed a limited preservation temperature (−2.5~−3 °C) and selective sample validation, including composition, presence of hard solid-like bone, and chemical state of proteins [1,11]. Still, techniques to stabilize supercooling condition of foods are required.

As a similar concept of subzero temperature preservation, the effect of superchilling preservation on the physicochemical properties of foods has been reported. Superchilling shares a preservation temperature (1–2 °C below the initial freezing point of foods) similar to that of supercooling (in case of meat −2~−4 °C), but the former allows the phase transition of foods, which must be prevented from the latter [12]. It was reported that superchilling preservation could provide better quality of foods than normal chilling conditions because it enabled minimization of microbial and chemical actions and did not require any process time for thawing [4,13,14]. On the other hand, some superchilling investigations reported negative aspects, such as tissue damage and high drip loss, which are typically shown in improperly frozen foods, and the deteriorations were more intense with the preservation period [15,16]. Nevertheless, superchilling has the potential application of a subzero temperature preservation technique due to its simple and convenient operation compared to supercooling. However, the quality characteristics of foods preserved by supercooling and superchilling have never been compared.

Korean Hanwoo is regarded as highly expensive premium beef breed but preferred meat by consumers. Beef requires a relatively long aging period compared to pork or poultry after slaughtering. In addition, vacuum packaging is not suitable for beef loin to maintain bright red color during retail displays [2]. In home chillers, therefore, the shelf life of beef loins is highly limited to a few days [1]. Consequently, this study investigated the effects of superchilling and supercooling on the freshness and physicochemical properties of Hanwoo beef loin for moderate storage periods.

## 2. Materials and Methods

### 2.1. Sample Preparations

Six sliced beef loins (Korean brown Hanwoo 1+ grade, 1.5 cm thickness, pH 5.61–5.93) were randomly purchased at 72–96 h postmortem from a local market (Seoul, Korea). From each loin, two rectangular slabs were sampled by avoiding the central connective tissue and were weighed (~150 g). The sample slabs were individually placed on a polystyrene tray and wrapped using a low-density polyethylene film, and the packaged samples were randomly divided into four groups (three packages each). One package from each group was attached to a T-type thermocouple on the packaging surface of the sample to avoid the thermocouple-induced sample freezing [1]. All chemicals used in this study were of analytical grade.

### 2.2. Low-Temperature Preservation

**Figure 1:**
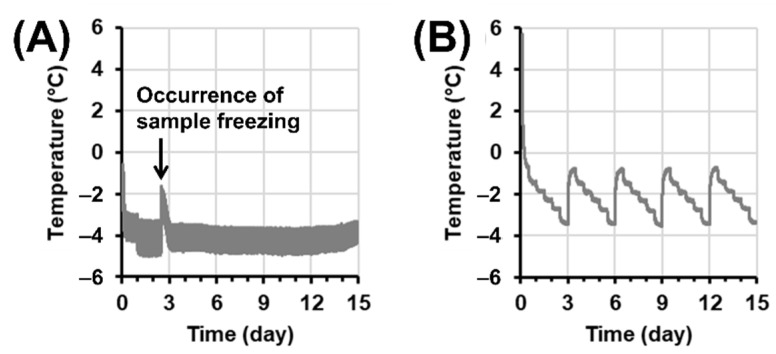
Representative time–temperature profiles of (**A**) superchilling and (**B**) supercooling preservation of beef loins.

One group was selected as a fresh control (no storage), and the other group was placed in a 4 °C home appliance refrigerator for chilling treatment. For subzero temperature preservation, a programmable cold incubator (FMU-053I, Fukushima Industries Corp., Osaka, Japan) was applied. The superchilling group was cooled in ice for 1 h and placed into an incubator, in which the temperature was maintained at −3.5 °C. With monitoring the temperature profile for 24 h, the preservation temperature was decreased to −4 °C if the meat sample did not undergo freezing in 24 h (Figure 1A). For supercooling preservation, a modified stepwise cooling algorithm of Park et al. (2021) [1] was applied. Sample packages were put into an expanded polystyrene (EPS) box (10 mm in thickness) to minimize temperature fluctuation of the samples and placed into the incubator, of which the temperature was set to −1 °C. At every 12 h interval, the setting temperature was decreased by 0.5 °C, and the temperature was again recovered to −1 °C after the end of the −3.5 °C stage. This procedure was repeated for a 15-day preservation period (Figure 1B). During preservation, the temperature of each treatment was monitored by connecting the thermocouples to a data logger (Yokogawa Co., Tokyo, Japan). After preservation, samples were aseptically depackaged and weighed, and 2 g from each sample slab was taken for microbial analysis. After taking surface color, the remaining portions of samples were further divided by ca. a 100 g portion was used for the thermal process, and the rest of the sample was used for chemical analyses. For experimental replication, the whole procedure was repeated three times on different days using a new batch of beef loin slices (*n* = 3).

### 2.3. Total Aerobic Count (TAC)

An aseptically taken 2 g sample was suspended in 18 mL sterile saline for 1 min using a stomacher (WS-400, Shanghai Zhisun Equipment Co., Ltd., Shanghai, China). After serial dilutions, 1 mL of sample was spread on a Petri film (aerobic count plate, 3 M Co., St. Paul, MN, USA) and incubated at 37 °C for 48 h. Films with 30–300 colonies were selected, and TCA was expressed as log colony forming unit (CFU)/g sample.

### 2.4. Total Volatile Basic Nitrogen (TVB-N)

The TVB-N of beef loin was measured by Park [1]. Meat (5 g taken from random position) was homogenized with 20 mL distilled water using a stomacher (WS-400, Shanghai Zhisun Equipment Co. Ltd., Shanghai, China) and filtered through filter paper (Whatman No. 2, GE Healthcare Life Science, Buckinghamshire, UK). The filtrate (1 mL) was transferred to the outer ring of a Conway dish, whereas 1 mL of 0.01 N H_3_BO_3_ and 100 μL of indicator solution (0.066% methyl red and 0.066% bromocresol green in ethanol, *w*/*v*) were loaded into the inner ring. After adding 1 mL of saturated K_2_CO_3_ solution into the outer ring, the Conway dish was sealed and incubated at 37 °C for 2 h. TVBN was titrated by 0.02 N H_2_SO_4_ until the color of the mixture in the outer ring changed from dark green to scarlet and calculated as

TVB-N (mg/100 g) = ((*A* − *B*) × *F* × 28.014)/*S* × 100 (1)

where *A* is the titer of the sample (mL), *B* is the titer of the blank (mL), *F* is a factor of 0.02 N H_2_SO_4_, and *S* is the weight of the sample (g).

### 2.5. Thioabarbituric Acid Reactive Substances (TBARS)

The TBARS of beef loin was determined by Witte [17] with minor modifications [1]. Briefly, a 5 g sample taken from random position was homogenized with 45 mL distilled water for 1 min and filtered through filter paper (Whatman No. 1, GE Healthcare Life Science, Buckinghamshire, UK). A 0.5 mL filtrate was mixed with 4.5 mL of TBA solution consisting of 0.25 M HCl, 15% (*w*/*v*) trichloroacetic acid and 0.375% (*w*/*v*) TBA reagent. After heating in a 95 °C water bath for 15 min, the mixture was centrifuged at 3000× *g* for 10 min at 4 °C, and the absorbance of the supernatant was measured at 535 nm. TBARS was calculated using a standard curve with malondaldehyde (MA) as a standard and expressed in mg MA/kg.

### 2.6. Instrumental Color

The CIE color parameters of beef loin were measured using a color reader (CR-10, Konica Minolta Sensing, Tokyo, Japan) calibrated with a standard white board. CIE L*, a* and b* were recorded as indicators of lightness, redness, and yellowness, respectively. The color was taken from the three random surfaces of each loin and averaged.

### 2.7. Drip Loss and Cooking Loss

Drip losses of three beef loins of each treatment were estimated based on the percent ratio of weight before and after preservation. Each 100 g sample was weighed and separately placed in a plastic bag. The samples were immersed and heated in a 75 °C water bath (working volume of 302 × 240 × 150 mm) for 30 min and cooled in ambient conditions for 30 min. Cooking loss was estimated by the percent ratio of weight before and after cooking.

### 2.8. Texture Profile Analysis (TPA)

After determination of cooking loss, the cooked samples were cut into 1 cm cubes. The TPA of each sample cut was measured using a texture analyzer (CT3, Brookfield Engineering Lab., Stoughton, MA, USA) equipped with a cylindrical probe (TA3/100, Brookfield Engineering Labs Inc.). A double compression cycle was applied to each cube under the conditions of 5 g trigger load, 50 mm/min test speed, and 80% compression. The TPA of each treatment was tested using 12 cubes and averaged.

### 2.9. Statistical Analysis

To compare the main effect (preservation method) on meat qualities, a completely randomized design was adopted. Averaged data from three of each experimental replication were analyzed by one-way analysis of variance (ANOVA) using SPSS software (ver. 24, IBM, Armonk, NY, USA). When the main effect was significant (*p* < 0.05), Duncan’s multiple range test was conducted as a post hoc procedure. For multivariate data analysis, principal component analysis (PCA) was conducted using XLSTAT (version 2021.4.1, Addinsoft Inc., New York, NY, USA).

## 3. Results and Discussion

### 3.1. Comparison of Preservation Conditions

In this study, all superchilled samples were not frozen within 24 h and were hence applied to −4 °C from 2 days (Figure 1A). Temperature during superchilling showed a fluctuation of ±0.9 °C; however, it should be noted that the temperature was measured on the surface of the sample package. The mean temperature of superchilling was −3.9 °C, and beef was frozen at 2.4, 4.2, and 5.8 days from experimental trials. Because the temperature during superchilling was evaluated on random samples, the time at which freezing occurred in individual samples was unpredictable.

**Figure 2:**
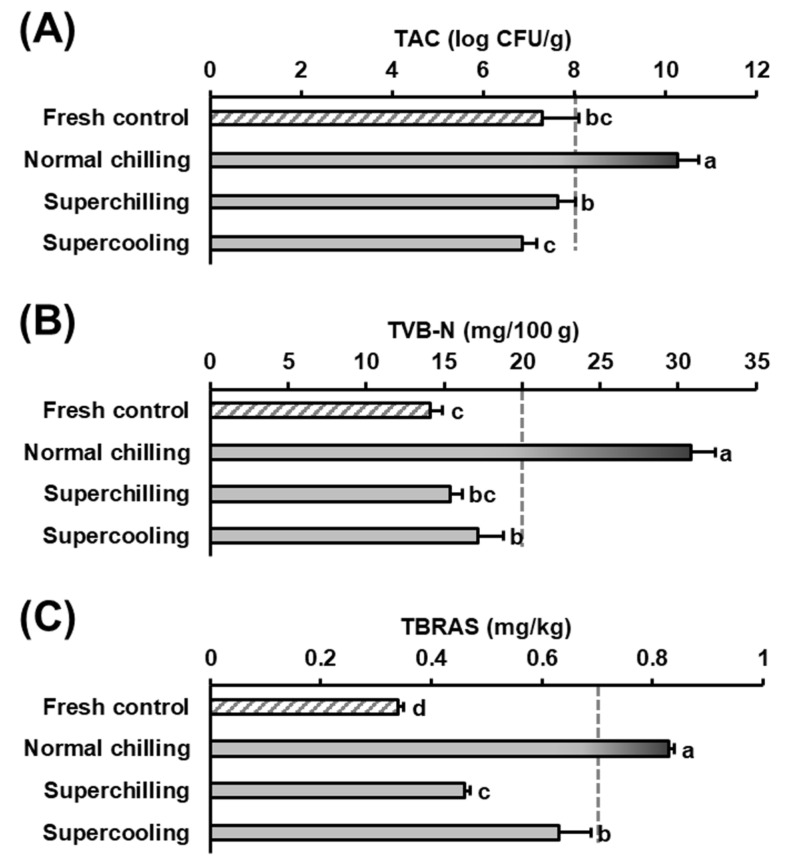
Comparisons of (**A**) total aerobic count (TAC), (**B**) total volatile basic nitrogen (TVB-N), and (**C**) thiobarbituric acid reactive substances (TBARS) of beef loins after 15 days of preservation. Bars indicate standard deviations (*n* = 3).

For supercooled beef, there was no evidence of meat freezing for the whole storage period (Figure 2B). Based on a preliminary study, beef loin was more stable for supercooling preservation than pork or fish; hence, the present study adopted −3.5 °C as the lowest temperature, which was lower than previous investigations [1,5]. The temperature of the supercooling group showed a limited fluctuation due to the utilization of an EPS box. The mean temperature of the whole storage period of supercooling was −2.1 °C, which was also comparably lower than the −1.5 °C of a previous investigation [1].

### 3.2. Freshness Indicators

Both superchilling and supercooling prevented or minimized microbial growth during 15 days of preservation (Figure 2A). Compared to 7.29 log CFU/g for the control beef, the chilled treatment showed 10.3 log CFU/g for the highest TAC among the treatments (*p* < 0.05). Meanwhile, the TAC of the superchilling and supercooling treatments ranged from 6.85–7.95 log CFU/g, showing no or a slight increase in TAC compared to the control. Based on the International Commission on Microbiological Specifications for Foods, the freshness criteria of meat cut are less than 6~7 log CFU/g, whereas meat with TAC greater than 8~9 log CFU/g is regarded as inedible [18,19]. In this study, beef loin was purchased after 3~4 days of retail display, which would account for the slightly higher TAC of the control than the freshness criteria, and the beef meat completely lost its edibility within 15 days of normal chilled storage. Alternately, little change in the TAC of both superchilling and supercooling preservation was likely due to the applied low preservation temperature. According to Park [1], stepwise supercooling preservation enabled us to extend the highly fresh condition of mackerel twice as long as chilling at 2 °C. In the present study, subzero temperature preservations were effective in reducing the microbiological risk of beef, regardless of the type of preservation.

The pattern of TAC was also found in the TVB-N of beef loin (Figure 2B). Compared to the 14.1 mg/100 g control, the chilled group showed significantly greater TVB-N (*p* < 0.05) and reached 30.8 mg/100 g after 15 days. However, both subzero temperature treatments showed 15.4–17.2 mg/100 g of TVB-N after storage. The TVB-N of the supercooling treatment was greater than that of the control (*p* < 0.05), possibly due to air-containing wrap packaging. However, there was no difference in TVB-N between the superchilling treatment and the control which resulted from the occurrence of beef freezing. Generally, meat cut with TVB-N below 20 mg/100 g is regarded as one of the important freshness criteria [1], and both subzero temperature treatments (superchilling and supercooling) acceptably extended the meat freshness for more than 15 days.

The preservation techniques showed a clear impact on lipid oxidation of beef loin (Figure 2C). Compared to 0.34 mg MA/kg TBARS in the control, all preservation treatments showed significantly higher TBARS (*p* < 0.05). In particular, the chilled group had 0.83 mg MA/kg TBARS, which already exceeded the maximum limit of 0.7 mg MA/kg [1]. It was reported that beef was sensitive to lipid oxidation among animal species due to the large amount of heme pigment [20]. Although the supercooling group also showed 0.63 mg MA/kg greater TBARS than the 0.46 mg MA/kg superchilling treatment (*p* < 0.05), the TBARS of the supercooling treatment remained fresh (< 0.7 mg MA/kg). Therefore, this study indicated that both superchilling and supercooling were effective in maintaining beef freshness for more than 15 days of storage. This advantageous effect mainly resulted from the relatively lower preservation temperature than normal chilled storage (4 °C). Meanwhile, superchilling could maintain the freshness of beef loin compared to supercooling, which was possibly due either to a lower mean temperature or to the occurrence of freezing. In particular, freezing prevents biochemical changes in meat during storage. Nevertheless, the freshness parameters evaluated in this study reflected that supercooling was also acceptable during the tested period (15 days).

### 3.3. Instrumental Color

After chilled storage for 15 days, a significant decrease in a* was observed (*p* < 0.05), whereas L* and b* did not differ from the fresh control (Table 1). The color change of the chilled treatment was evidenced by oxidation of myoglobin, and the intensity of browning discoloration could be identified visually (Figure S1). Alternately, both subzero temperature treatments exhibited significantly high L* and b* values (*p* < 0.05) without difference in a* value compared to fresh control. Although the browning of meat was not detected in superchilling and supercooling treatments, the superchilling group showed a lower b* than that of the supercooling group (*p* < 0.05). In this study, all meat samples were wrapped using an oxygen permeable film, and oxygenation of myoglobin would predominantly occur during the early storage period of all treatments. The oxidation rate of myoglobin was temperature dependent, and supercooling minimized the oxidation of myoglobin compared to the chilled treatment [21]. Similar results were also found in mackerel fillets, pork loins and chicken breasts stored under supercooling [1,10]. Meanwhile, superchilling treatment initiated freezing for at least 2 days of storage, and hence, freezing would prevent oxymyoglobin from chemical oxidation [22], which likely affected the best color stability of superchilling treatment even after 15 days of storage. However, it was possible that the superchilling treatment was kept at a lower temperature than supercooling, which could also affect the color stability of the superchilled group. Based on the instrumental color parameters, it was clear that subzero temperature preservation was effective in maintaining a fresh meat-like color on a week-scale. However, it was expected that superchilling would have a better impact on the color stability of beef loin than supercooling if the storage periods were extended to the month scale.

### 3.4. Drip Loss and Cooking Loss

The drip loss and cooking loss of beef loins stored under varying conditions for 15 days are compared in Table 2. Compared to the 2.93% drip loss of the chilled treatment, the superchilling group exhibited a 3.37% significantly higher drip loss (*p* < 0.05). Meanwhile, the supercooling treatment showed 1.68% of the lowest drip loss among the treatments (*p* < 0.05). The latter supercooling result was consistent with the literature, where the supercooled fish, pork, or chicken had a much lower drip loss than the chilled treatments [1,10]. Interestingly, these studies compared the physicochemical properties of supercooling treatment with frozen counterparts and indicated that drip loss of frozen samples was higher than supercooling treatment but significantly lower than chilled sample after 2 weeks of storage. It was obvious that decreasing the preservation temperature was an effective way to minimize the drip loss of muscle foods; nevertheless, the higher drip loss of the superchilling treatment than supercooling indicated that physical tissue damage had a greater impact on drip loss than preservation temperature. In particular, the superchilling temperature (−3.9 °C) was relatively higher than the normal freezing temperature (−18 °C), and a superchilling environment promoted ice recrystallization once the sample was frozen [23]. Eventually, high drip loss of superchilled beef would be more pronounced with extending storage period, and it could be a major disadvantage of superchilling preservation.

Cooking loss of beef loin decreased from 27.3% of control to 18.4% after chilled storage for 15 days (*p* < 0.05), whereas those of both superchilling and supercooling treatment did not differ from control and ranged to 26.6–28.0%. It was known that cooking loss was related to the chemical state of meat protein. During storage, lipid oxidation manifested as protein oxidation resulting in the loss of water-binding properties, thereby showing higher cooking loss [24]. As shown in freshness indicators, however, chilled beef showed excessive microbial counts among treatments. The action of microbial action caused the degradation of muscle proteins, which increased the water-binding property of the proteins [25], which probably accounted for the latter decrease in cooking loss of beef loin. Alternately, subzero temperature could prevent or delay the microbial and chemical changes of meat during 15 days of storage, which was also evidenced in freshness indicators.

### 3.5. TPA

Supercooling treatment maintained the textural properties of fresh beef loin, for which the TPA parameters did not differ from those of the control (Table 3). Meanwhile, both the chilling and superchilling treatments exhibited different textural features from the control, but their textural features were different from each other. The chilling treatment showed lower hardness and higher cohesiveness than the control (*p* < 0.05). In general, meat becomes tender with storage periods due to microbial actions and intrinsic proteases. In addition, the development of sticky texture is found in muscle foods after excessive chilled storage [26]. Nevertheless, the chilled treatment did not show differences in springiness and chewiness with those of the control. Alternately, superchilling treatment showed significantly higher hardness, cohesiveness, and chewiness than those of control (*p* < 0.05). There is a lack of information regarding the tenderness of superchilled meat because varying superchilled conditions were compared with conventional chilled meat [4]. However, it seemed that the tenderness of superchilled meat was dependent on the occurrence of meat freezing. Based on Ding [27], pork stored at −1 °C exhibited fewer changes in shear force than those stored at −2 °C or −3 °C, since ice crystals did not form in the former. Based on the results, it was unclear whether superchilling had an advantage in maintaining the quality of beef loin. As revealed by the physicochemical parameters, the characteristics of superchilled beef loin were probably involved in freezing during storage. In this case, superchilling is regarded as a type of freeze storage adopting a relatively high preservation temperature (approximately −4 °C). Qian [23] stored beef under varying subfreezing storage temperatures (−6~−12 °C) and indicated that quality and shelf life depended on storage temperature. As a result, superchilling effectively extended the shelf life of meat compared to normal chilling conditions, but the effects on beef quality were not as pronounced as supercooling.

### 3.6. PCA

**Figure 3:**
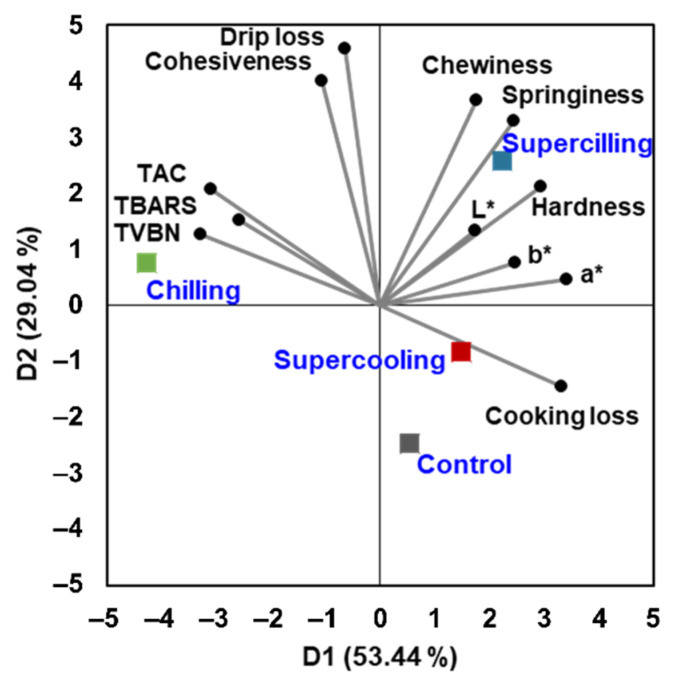
Principal component analysis of dependent variables and preservation treatments of beef loin.

In this study, two component dimensions accounted for 82.48% of the data variation (Figure 3). The first dimension (D1), which explained 53.44% of the data variation, was related to freshness indicators (TAC, TVB-N and TBARS), a* and cooking loss. From the first dimension, superchilling and supercooling treatments were grouped with the control, whereas chilling treatment was clearly separated from the group. The second dimension (D2) explained 29.04% of the data variation and was related to drip loss and TPA parameters (cohesiveness and chewiness). Based on D2, superchilling was clearly separated from the control, whereas supercooling was placed with the control. Based on the results, subzero temperature was suitable to extend the freshness of beef loin for 15 days regardless of freezing. However, the occurrence of freezing affected the drip loss and texture of beef loin, which diminished the advantage of superchilling. Finally, supercooling was recommended for preservation of beef loin from freshness and quality viewpoints.

## 4. Conclusions

It was clear that the freshness of meat could be extended by decreasing the preservation temperature, and both superchilling and supercooling effectively minimized microbial growth and biochemical actions in beef loin for 15 days of preservation. Nevertheless, the generation of stable supercooling conditions is not obtained by simple techniques, and superchilling has the potential to replace the subzero temperature preservation of meat. However, superchilling showed drawbacks in terms of the qualities of beef loins even though it was conducted at a lower temperature than supercooling treatment. To prevent the occurrence of freezing, the superchilling temperature has to be selected to be around the freezing point of meat (around −1 °C), but it is not useful to replace supercooling preservation. In addition, unpredictable freezing of superchilling limits the standardization of superchilling preservation. Therefore, this study demonstrated that supercooling was a potential technique for the preservation of beef loin while retaining its original quality, and additional best conditions for superchilling preservation warrant further exploration.

## Figures and Tables

**Table 1:** Comparisons of CIE color parameters of beef loins after 15 days of preservation.

Treatments	Fresh Control	Normal Chilling	Superchilling	Supercooling
L*	34.6 ± 7.81 ^c^	36.9 ± 1.86 ^bc^	41.5 ± 3.70 ^ab^	46.4 ± 4.49 ^a^
a*	10.6 ± 3.02 ^a^	5.22 ± 0.95 ^b^	12.9 ± 1.70 ^a^	10.5 ± 2.36 ^a^
b*	10.2 ± 2.18 ^c^	9.80 ± 0.89 ^c^	12.0 ± 0.72 ^b^	13.4 ± 1.05 ^a^

Means with different superscripts among the same column are significantly different (*p <* 0.05).

**Table 2:** Comparisons of drip loss and cooking loss of beef loins after 15 days of preservation.

Treatments	Fresh Control	Normal Chilling	Superchilling	Supercooling
Drip loss (%)	ND	2.93 ± 0.67 ^b^	3.37 ± 0.51 ^a^	1.68 ± 0.20 ^c^
Cooking loss (%)	27.3 ± 1.84 ^a^	18.4 ± 0.49 ^b^	26.6 ± 2.52 ^a^	28.0 ± 3.70 ^a^

Means with different superscripts among the same column are significantly different (*p* < 0.05).

**Table 3:** Comparisons of texture profile analysis (TPA) parameters of beef loins after 15 days of preservation.

Treatments	Fresh Control	Normal Chilling	Superchilling	Supercooling
Hardness (N)	61.5 ± 8.28 ^b^	51.1 ± 5.06 ^c^	75.7 ± 10.5 ^a^	60.7 ± 7.98 ^b^
Cohesiveness	0.26 ± 0.04 ^b^	0.31 ± 0.04 ^a^	0.32 ± 0.04 ^a^	0.24 ± 0.04 ^b^
Springiness (mm)	2.73 ± 0.22	2.74 ± 0.55	3.00 ± 0.32	2.88 ± 0.34
Chewiness (mJ)	44.3 ± 15.2 ^b^	43.0 ± 8.56 ^b^	74.2 ± 17.9 ^a^	41.9 ± 11.7 ^b^

Means with different superscripts among the same column are significantly different (*p* < 0.05).

## Data Availability

All data generated or analyzed during this study are included in this published article.

## Supplementary Materials

The following supporting information can be downloaded at: https://www.mdpi.com/article/10.3390/foods11182729/s1, Figure S1: Comparisons of appearance of beef loins before and after 15 days of preservation.
