# Supplementary figures and images for: Comparison of Superchilling and Supercooling on Extending the Fresh Quality of Beef Loin
Source: Foods. 2022 Sep 6;11(18):2729. doi: 10.3390/foods11182729 (PMC9498017; doi:10.3390/foods11182729)

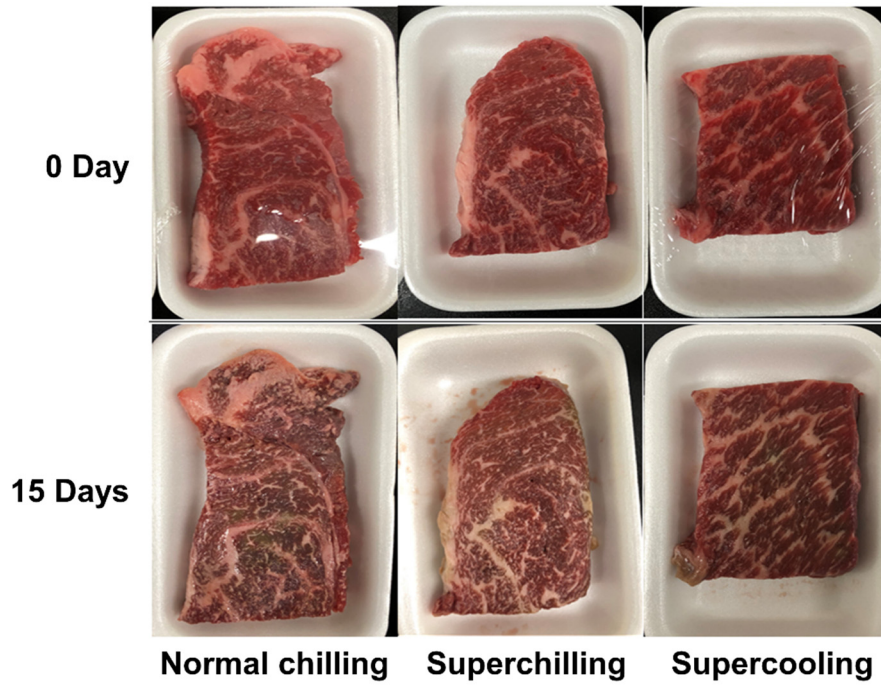

**Figure S1.** Comparisons of appearance of beef loins before and after 15 days of preservation.

Supplement: Supplementary file 1 [file foods-11-02729-s001.zip › foods-1887751-supplementary.pdf]
